# Effects of Resistance Training on Matrix Metalloproteinase Activity in Skeletal Muscles and Blood Circulation During Aging

**DOI:** 10.3389/fphys.2018.00190

**Published:** 2018-03-12

**Authors:** Ivo V. de Sousa Neto, João L. Q. Durigan, Vinicius Guzzoni, Ramires A. Tibana, Jonato Prestes, Heloisa S. Selistre de Araujo, Rita de Cássia Marqueti

**Affiliations:** ^1^Graduate Program of Sciences and Technology of Health, University of Brasilia, Brasília, Brazil; ^2^Department of Physiological Sciences, Federal University of São Carlos, São Carlos, Brazil; ^3^Graduate Program of Physical Education, Federal University of Mato Grosso, Cuiabá, Brazil; ^4^Graduate Program of Physical Education, Catholic University of Brasilia, Brasília, Brazil

**Keywords:** aging, extracellular matrix, muscle remodeling, matrix metallopeptidase, exercise training

## Abstract

Aging is a complex, multifactorial process characterized by the accumulation of deleterious effects, including biochemical adaptations of the extracellular matrix (ECM). The purpose of this study was to investigate the effects of 12 weeks of resistance training (RT) on metalloproteinase 2 (MMP-2) activity in skeletal muscles and, MMP-2 and MMP-9 activity in the blood circulation of young and old rats. Twenty-eight Wistar rats were randomly divided into four groups (*n* = 7 per group): young sedentary (YS); young trained (YT), old sedentary (OS), and old trained (OT). The stair climbing RT consisted of one training session every 2 other day, with 8–12 dynamic movements per climb. The animals were euthanized 48 h after the end of the experimental period. MMP-2 and MMP-9 activity was measured by zymography. There was higher active MMP-2 activity in the lateral gastrocnemius and flexor digitorum profundus muscles in the OT group when compared to the OS, YS, and YT groups (*p* ≤ 0.001). Moreover, there was higher active MMP-2 activity in the medial gastrocnemius muscle in the OT group when compared to the YS and YT groups (*p* ≤ 0.001). The YS group presented lower active MMP-2 activity in the soleus muscle than the YT, OS, OT groups (*p* ≤ 0.001). With respect to active MMP-2/9 activity in the bloodstream, the OT group displayed significantly reduced activity (*p* ≤ 0.001) when compared to YS and YT groups. In conclusion, RT up-regulates MMP-2 activity in aging muscles, while down-regulating MMP-2 and MMP-9 in the blood circulation, suggesting that it may be a useful tool for the maintenance of ECM remodeling.

## Introduction

Aging is a complex, multifactorial process characterized by the accumulation of deleterious changes at the cellular level in multiple systems, including skeletal muscles, which promotes the loss of muscle mass and deterioration of physiological function (Joanisse et al., 2016). Among these deleterious changes, are the increase in collagen concentration and the emergence of cross-links between collagen fibers in the extracellular matrix (ECM) (Kragstrup et al., 2011; Doria et al., 2012). The ECM surrounding skeletal muscle fibers provides structural support, protection, and maintenance of the functional integrity of skeletal muscle through several mechanisms, including the transmission of lateral strength between fibers and fascicles and the passive elastic response in the muscle contraction process (Carmeli et al., 2004).

The modulation of function is controlled by matrix metalloproteinases (MMPs). MMPs are a family of zinc- and calcium-dependent enzymes that promote the degradation and synthesis of ECM components, such as collagen, proteoglycans and glycoproteins during normal and pathological tissue remodeling (Marqueti et al., 2008). The ECM remodeling in skeletal muscles depends on the MMP action, specifically MMP-2 (gelatinase A) and MMP-9 (gelatinase B), which promote morphogenesis, and angiogenesis (Carmeli et al., 2004; Deus et al., 2012). These enzymes stimulate the release of local growth factors in skeletal muscles (Heinemeier et al., 2009), the proliferation, differentiation and migration of satellite cells following damage (Kjaer, 2004) and local connective tissue homeostasis (Chen and Li, 2009). In addition, MMPs are related to the modulation of inflammatory processes at various levels, such as bioavailability and activity of inflammatory cytokines, as well as regulating the survival and evasion of inflammatory cells (Nissinen and Kahari, 2014; Smigiel and Parks, 2017). For example, it has been demonstrated that MMPs can orchestrate the transmigration of inflammatory cells from the blood circulation to the site of inflammation in skeletal muscle by processing ECM components (Parks et al., 2004; Nissinen and Kahari, 2014).

Recent observations provide evidence that gelatinase activity in the blood circulation can be considered mediators of inflammation and provide important information about inflammatory states during the aging process and pathological conditions (Giannakos et al., 2016; Nascimento et al., 2016; Lo Presti et al., 2017; Smigiel and Parks, 2017). High MMP-2 and MMP-9 activity may be related to immunity, tumor progression or apoptosis, rheumatoid arthritis, obesity and may play a role in cardiovascular disease diagnoses (Nascimento et al., 2015, 2016; Giannakos et al., 2016). However, the findings in the blood circulation may not be an accurate reflection of skeletal muscle adaptations since systemic MMPs reflect the balance between their release from many cell types into the bloodstream and their removal from circulation (Nascimento et al., 2015; Lo Presti et al., 2017; Sousa Neto et al., 2017).

In this context, it has been demonstrated that an imbalance in the turnover of ECM components during aging may result in unfavorable remodeling processes and can change biochemical adaptations, including MMP activity (Payne, 2006; Kragstrup et al., 2011). The inflammatory profile of aging down-regulates hypertrophy signaling, which contributes to sarcopenia (Jang and Van Remmen, 2011; Joanisse et al., 2016). Additionally, the aging process has been associated with increasing body fat which favors functional decline and increases obesity-related cardiovascular morbidity-mortality (Souza et al., 2014). Consequently, further investigations are necessary to determine strategies to prevent or attenuate these negative effects on muscle physiology and immunity.

Nevertheless, resistance training (RT) modulates MMP activity. Souza et al. (2014) and Prestes et al. (2009) showed an increase in MMP-2 activity induced by RT (12 weeks, 3 times per week) in an obesity model and hypoestrogenism in rat skeletal muscles, which indicates that mechanical loading exercise exerts important regulatory roles in ECM homeostasis and the remodeling of muscle fibers and local connective tissue. In addition, previous studies have demonstrated that mechanical load exercise promotes positive adaptations in the ECM in the muscle and partially reverses the deleterious effects of aging through increased collagen I and III turnover (Kragstrup et al., 2011; Wood and Brooks, 2016; Zotz et al., 2016). Finally, RT can be a helpful tool to control MMP-2 and MM-9 activity in the circulation and to control the inflammatory balance (Nascimento et al., 2015). Although evidence indicates that RT favorably alters the ECM remodeling in skeletal muscles, the effects of RT on MMP activity in the skeletal muscles and bloodstream of old rats has not been assessed. This is information would be valuable to elucidate possible adaptive mechanisms induced by RT.

Thus, the aim of the present study was to investigate the effects of 12 weeks of RT on MMP-2 activity in skeletal muscles and MMP-2 and MMP-9 activity in the blood circulation of young and old rats. The novelty of this study was the influence of exercise training on MMP activity in different skeletal muscles and the blood circulation during the aging process. Our hypothesis is that RT up-regulates MMP-2 activity in skeletal muscles, regardless of the aging process, while down-regulating MMP-2 and MMP-9 in the blood.

## Materials and methods

### Animals

All procedures were conducted in accordance with the U.S. Guide for the Care and Use of Laboratory Animals (National Research Council, 1998). The research protocol received approval from the Ethics Committee on Animal Experimentation from the Federal University of São Carlos, SP, Brazil (process: 056/2010). Twenty-eight male Wistar rats (*Ratus norvegicus albins*) aged three to 21 months were used at the beginning of the RT protocol. The classification of “young” and old “animals” was determined according to Quinn (2005). The animals were placed in collective cages (maximum four rats per cage) with water and standard rodent chow (Purina®, Descalvado, São Paulo, Brazil) available ad libitum, and exposed to constant temperature (22 ± 2°C) and light cycle (12:12 h light-dark cycle). The animals and tissues were weighed in grams using a digital scale (Filizola®, São Paulo, Brazil) before and after RT.

### Experimental groups

Rats were randomly divided into four groups (seven animals per group): young sedentary (YS), young trained (YT), old sedentary (OS), and old trained (OT) rats. The present study design is a data analysis of a previous project (Ribeiro et al., 2017).

### Resistance training

#### Familiarization

The rats were adapted to a RT protocol of climbing a vertical ladder (1.1 m; 0.18 m, 2-cm grid, 80° incline) with no weight on the load apparatus for two non-consecutive days (48-h-rest interval). The load apparatus was fixed to the tail by wrapping its proximal portion with a self-adhesive foam strip. Rats were placed at the bottom of the ladder and familiarized with climbing. Finger pinching was used on the animal's tail as a stimulus to initiate climbing. At the top of the ladder, the rats reached a chamber where they rested for 2 min. This procedure was repeated until they would voluntarily climb the ladder for three consecutive turns without stimulus.

#### Determination of maximum carrying capacity

Two days after the familiarization procedure, each animal was evaluated to establish the maximum carrying load, which consisted of 4–9 ladder climbs with progressively heavier loads interspersed by a 120 s interval between each attempt. The initial climb was performed with 75% of the animal's body mass. Upon successful completion of this load, an additional 30-g weight was added to the load apparatus. The highest load that the animal successfully carried through the entire length of the ladder was considered the rat's maximal carrying capacity for that training session. Failure was determined when the animal could not progress up the ladder after three successive stimuli to the tail.

#### Resistance training period

During the 12 weeks of RT, climbing sessions were performed 3 times per week (Monday, Wednesday, and Friday) during the afternoon (between 2:00 and 4:00 p.m.). The length of the ladder allowed the animals to make 8–12 dynamic movements per climb. The climbs consisted of carrying a progressive load of 65, 85, 95, and 100% of the maximum carrying capacity of each animal. RT sessions consisted of 5–8 movements per climb over 6–8 s. If a rat reached 100% of its carrying capacity, an additional 30-g load was added until a new maximum carrying capacity was determined with a maximum of eight climbs per session. The resting period between each climb was 2 min. This RT protocol was adapted from Hornberger and Farrar (2004). Training procedures have also been described elsewhere (Prestes et al., 2009; Souza et al., 2014; Sousa Neto et al., 2017).

### Euthanasia

To avoid the acute effects of RT, the animals were euthanized using an intraperitoneal injection of xylazine solution (12 mg/kg of body weight) and ketamine (95 mg/kg of body weight) 48 h after the end of the experimental period.

### Zymography

#### Muscles

After euthanasia, the lateral and medial gastrocnemius, flexor digitorum profundus and soleus were dissected and immediately washed with saline. The samples were weighed and frozen in liquid nitrogen, and stored in a freezer at −84°C for subsequent biochemical analysis. The tissue samples for extraction were treated as described elsewhere (Cleutjens et al., 1995). Frozen tissue (20 mg) was incubated in 1.0 ml extraction buffer (10 mM cacodilic acid pH 5.0, 0.15 M NaCl, 1 mM ZnCl^2^, 20 mM CaCl^2^, 1.5 mM NaN_3_, Triton X-100 0. 01% [v/v]) at 4°C for 24 h. After this period, the solution was centrifuged (10 min, 13,000 × g at 4°C).

#### Blood plasma

The blood specimens were removed from the inferior vena cava and mixed with 3.2% solution of sodium citrate in a volume of 9:1. After 15 min of centrifugation at 3,000 rpm, the supernatant was recovered. The samples containing 0.5 uL of plasma were added to 0.5 ul of SDS (8%) (v:v). Subsequently, they were placed in a vortex, and 10 uL of sample buffer without β-mercaptoethanol (reducing agent), containing SDS (20%) was added.

The samples were analyzed by electrophoresis in polyacrylamide gel containing 10% SDS and gelatin at a final concentration of 1 mg/ml. After electrophoresis, the gels were washed twice in 2.5% Triton X-100 to remove SDS and incubated in a substrate buffer (50 mM Tris-HCl pH 8.0, 5 mM CaCl^2^ and 0.02% NaN_3_) at 37°C for 20 h. The gels were stained with Coomassie Brilliant Blue R-250 (Bio -Rad) for 1 ½ h and destained with acetic acid, metanol and water. The gelatinolytic activity was visualized as clear bands in the stained gel. The gels were photographed with a 7.1 megapixel Canon Power Shot G6 and the average intensity of the bands were analyzed using the software Gene Tools. Pro (latent), intermediate and active bands were identified via standard techniques using molecular weight criteria (Marqueti et al., 2008; Sousa Neto et al., 2017).

### Statistical analysis

The results were expressed as the means ± the standard deviation (SD). Kolmogorov - Smirnov and Levene's tests were used to analyze the homogeneity of the variance. A two-way ANOVA (training vs. age) was used to compare body weight, muscle weight and MMP activity in skeletal muscles and blood plasma between groups. When a significant difference was detected, a Tukey post hoc test was applied to identify the differences. An alpha level of *p* ≤ 0.05 was considered significant. The sample size power for all variables (MMP-2 and MMP-9 activity) was verified post hoc using G^*^Power version 3.1.3 (Kiel University, Kiel, Germany), with alpha level = 0.05 and power (1–β) = 0.8. The software Statistica 6.1 (StatSoft Inc., Tulsa, OK, USA) was used for statistical analysis. In addition, GraphPad Prism 6.0 (San Diego, CA, USA) was also used for the graphics design.

## Results

### Body and skeletal muscle weights of experimental groups

Initial body weight was greater in old rats (OS and OT) when compared to young animals (YS and YT) (*p* = 0.01). With respect to body weight after the training period, the young groups displayed a significant increase (*p* = 0.01), while the old animals displayed a significant decrease (*p* = 0.01). Additionally, there was no statistically significant interaction between groups with respect to the weights of the lateral and medial gastrocnemius, flexor digitorum profundus, and soleus muscles (Table 1).

**Table 1:** Final body weight and muscle weight of experimental groups.

	**YS**	**YT**	**OS**	**OT**
Initial body weight (g)	295.6 ± 34.6	301.8 ± 30	508.3 ± 76.6^*^^†^	527.3 ± 75.9^*^^†^
Final body weight (g)	508.3 ± 85.6^a^	434 ± 52.7^a^	452 ± 86.4^a^	480.4 ± 66.5^a^
Gain (%)	57.8	44.4	−6.8	−15
Gastrocnemius medial weight (mg)	1.190 ± 0.17	1.121 ± 0.11	1.072 ± 0.10	0.976 ± 0.1
Gastrocnemius lateral weight (mg)	1.367 ± 0.19	1.238 ± 0.14	0.884 ± 0.16	1.211 ± 0.12
Flexor digitorum profundus weight (mg)	0.667 ± 0.06	0.690 ± 0.04	0.568 ± 0.08	0.707 ± 0.019
Soleus weight (mg)	0.279 ± 0.04	0.248 ± 0.02	0.237 ± 0.04	0.260 ± 0.02

The data are mean ± standard deviation. The experimental groups are represented as young sedentary (YS), young trained (YT), old sedentary (OS), and old trained (OT). Gain (%) was the difference between initial and final weight. Statistically significant differences compared to:

* YS,

† YT,

a *initial body weight, p < 0.05. (n = 7 per group)*.

### Effects of aging and RT on MMP-2 activity in skeletal muscles

**Figure 1:**
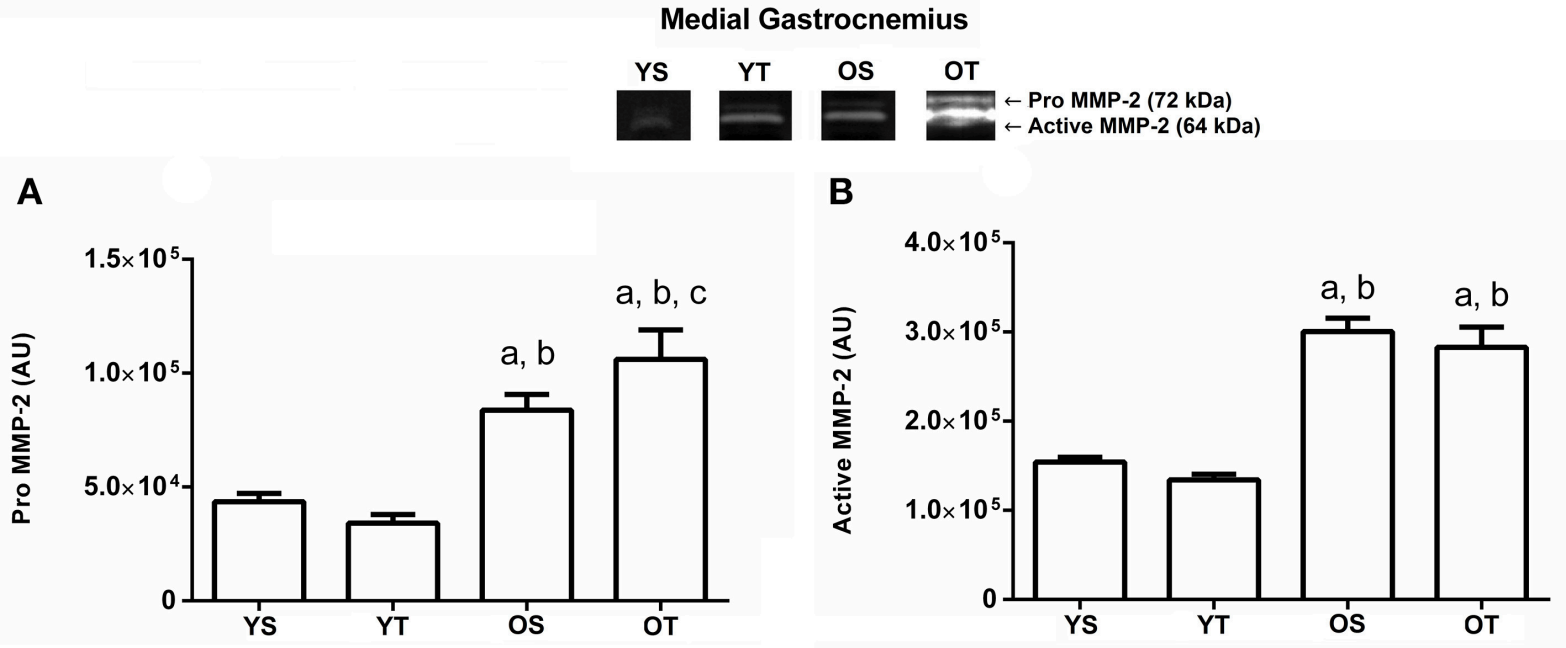
Optical densitometry of zymographic bands of MMP-2 in arbitrary units (AU) in the medial gastrocnemius muscle. The data are mean ± standard deviation. **(A)** represents Pro MMP-2 (72 kDa), **(B)**: represents active MMP-2 (64 kDa). The experimental groups were represented as young sedentary (YS) young trained (YT), old sedentary (OS) and old trained (OT). Statistically significant differences compared to: ^a^YS; ^b^YT; ^c^OS, *p* < 0.05. (*n* = 7 per group).

There was a statistically significant interaction between training and age for pro MMP-2 activity in the medial gastrocnemius muscle. The OS group displayed an increase compared to YS and YT groups. The OT group showed the highest activity values when compared to the other groups (*p* = 0.001; Figure 1A). Furthermore, the active MMP-2 activity is modified by age status, which showed an increased in the OS and OT groups when compared to YS and YT groups (*p* = 0.001; Figure 1B).

**Figure 2:**
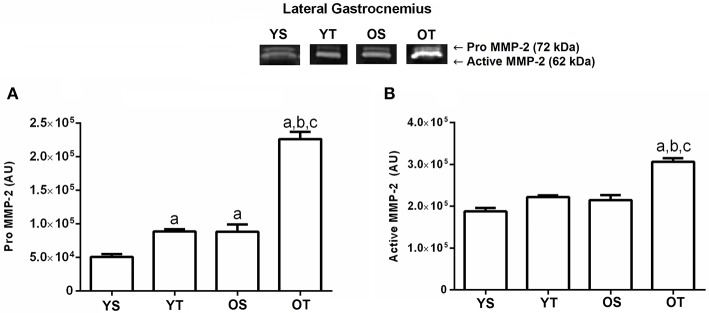
Optical densitometry of zymographic bands of MMP-2 in arbitrary units (AU) in the lateral gastrocnemius muscle. The data are mean ± standard deviation. **(A)** represents Pro MMP-2 (72 kDa), **(B)**: represents active MMP-2 (64 kDa). The experimental groups were represented as young sedentary (YS) young trained (YT), old sedentary (OS) and old trained (OT). Statistically significant differences compared to: ^a^YS; ^b^YT; ^c^OS, *p* < 0.05. (*n* = 7 per group).

Again, there was a significant interaction between training and age in pro MMP-2 activity in the lateral gastrocnemius muscle. The OS group demonstrated an increase compared with the YS group. Training significantly increased the pro MMP-2 activity in the YT (*p* = 0.01) and OT groups (*p* = 0.01) when compared to the YS and OS groups, respectively. The OT group displayed higher activity of MMP-2 when compared to the other groups (*p* = 0.01; Figure 2A). Additionally, the active MMP-2 activity increased significantly in the OT group when compared to the other experimental groups (*p* = 0.001; Figure 2B).

**Figure 3:**
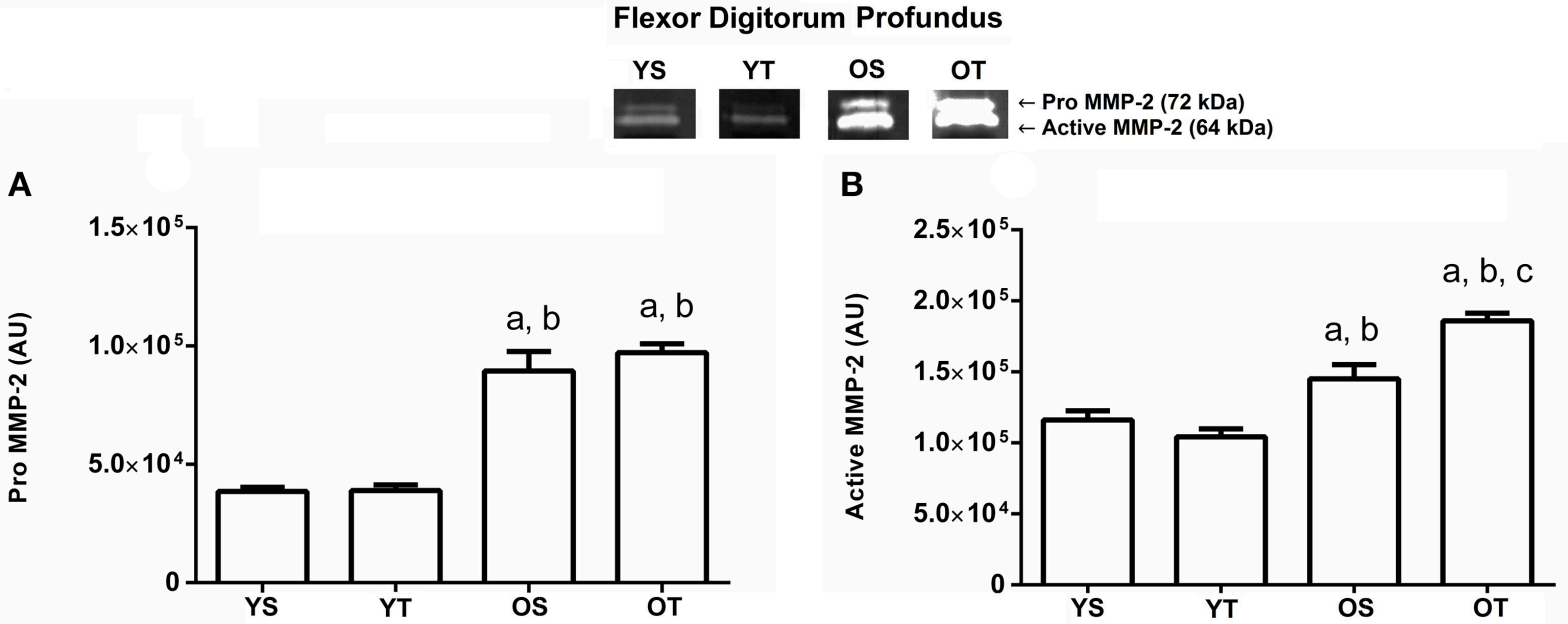
Optical densitometry of zymographic bands of MMP-2 in arbitrary units in the flexor digitorum profundus muscle. The data are mean ± standard deviation. **(A)** represents Pro MMP-2 (72 kDa), **(B)**: represents active MMP-2 (64 kDa). The experimental groups were represented as young sedentary (YS) young trained (YT), old sedentary (OS) and old trained (OT). Statistically significant differences compared to: ^a^YS; ^b^YT; ^c^OS, *p* < 0.05. (*n* = 7 per group).

The pro and active MMP-2 activity increased in both old groups (OS and OT) in the flexor digitorum profundus muscle when compared to the young groups (*p* = 0.04 and *p* = 0.001, respectively; Figures 3A,B). In addition, there was a statistically significant interaction between groups for the active MMP-2 form, indicating higher activity in the OS group when compared to the other experimental groups (*p* = 0.001; Figure 3B).

**Figure 4:**
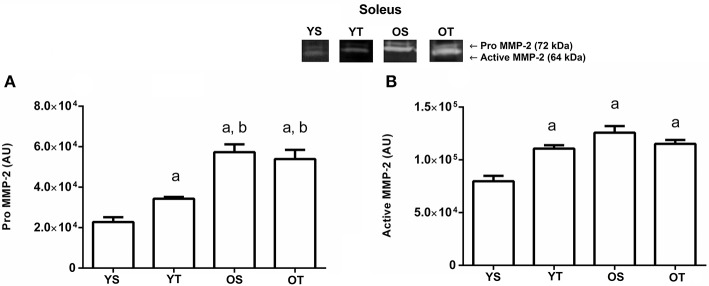
Optical densitometry of zymographic bands of MMP-2 in arbitrary units in muscle Soleus. The data are mean ± standard deviation. **(A)** represents Pro MMP-2 (72 kDa), **(B)** represents active MMP-2 (64 kDa). The experimental groups were represented as young sedentary (YS) young trained (YT), old sedentary (OS) and old trained (OT). Statistically significant differences compared to: ^a^YS; ^b^YT, *p* < 0.05. (*n* = 7 per group).

The YT group displayed a higher level of pro MMP-2 activity (*p* = 0.01) in the soleus muscle when compared to the YS group, and in OS and OT groups when compared to the YS and YT groups, respectively (*p* = 0.01; Figure 4A). All experimental groups increased (*p* = 0.001) the active MMP-2 activity when compared to the YS group, with no statistically significant interaction between training and age (*p*> 0.05; Figure 4B).

### Effects of aging and RT on MMP-2 and MMP-9 activity in blood circulation

**Figure 5:**
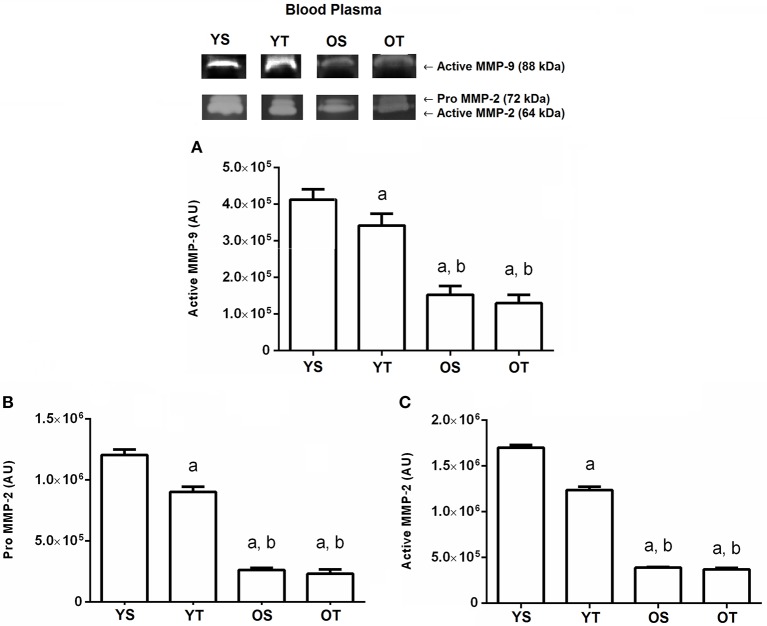
Optical densitometry of zymographic bands of MMP-2 and MMP-9 in arbitrary units in blood plasma. The data are mean ± standard deviation. **(A)** represents active MMP-9 (88 kDa), **(B)** represents Pro MMP-2 (72 kDa), **(C)**: represents active MMP-2 (64 kDa). The experimental groups were represented as young sedentary (YS) young trained (YT), old sedentary (OS) and old trained (OT). Statistically significant differences compared to: ^a^YS; ^b^YT, *p* < 0.05. (*n* = 7 per group).

Finally, the active MMP-9 activity in the bloodstream showed a significant reduction in YT (*p* = 0.001) when compared to the YS group. The active MMP-9 form reduced in the OS and OT groups when compared to the YS and YT groups, respectively (*p* = 0.001; Figure 5A). With respect to pro and active MMP-2 activity analysis in blood plasma, similar results were found. In the YT group, both forms of MMP-2 were reduced (*p* = 0.001) when compared to the YS group. The OS and OT groups also had reduced pro and active MMP-2 activity when compared to the YS and YT groups (*p* = 0.001), respectively (Figures 5B,C).

## Discussion

The objective of the present study was to investigate the effects of 12 weeks of RT on MMP-2 activity in skeletal muscles and MMP-2 and MMP-9 activity in the circulation of young and old rats. The results revealed that RT up-regulated MMP-2 activity in skeletal muscle of young and old rats. In addition, RT probably promoted MMP-2 recruitment from the circulation to skeletal muscles in response to demand and in favor of adequate local remodeling, which elucidates a decrease in MMP-2 and MMP-9 activity in old rats when compared with the young rats. The clinical relevance of our findings is based on evidence that the deleterious effects of aging promoted a loss of muscle mass and deterioration of physiological function in skeletal muscles, as well unfavorable tissue remodeling. In this context, a greater understanding of the RT-induced changes in MMP modulation in skeletal muscles is essential to prevent the age-induced decline in muscle function.

The motor performance in a stair-climbing exercise depends on several muscles. Each muscle analyzed in the present study has a different physiological response and biomechanical function which can promote distinct biochemical adaptations. It has been demonstrated that exercise induces an increase in MMP-2 activity in both type I and II fiber-predominant muscles, however, the predominance of glycolytic fibers favors an increase in MMP-2 activity (Hadler-Olsen et al., 2015). Previous studies demonstrated that the soleus and lateral gastrocnemius are muscles with a predominance of type I fibers (Delp and Duan, 1996; Staron et al., 1999). However, the medial gastrocnemius and flexor digitorum profundus have a predominance of type II fibers (Delp and Duan, 1996; Staron et al., 1999). In addition, aging promotes a conversion from fast to slow fibers due to the disuse of fast fiber motor units (Ibebunjo et al., 2013). These factors may explain the absence of differences in active MMP-2 activity between the soleus and medial gastrocnemius muscles in the OT group compared to YT and OS groups.

The results presented in the current study are in agreement with previous investigations that evaluated the influence of RT on MMP-2 activity in skeletal muscles of chronic disease models. For example, Souza et al. (2014) reported that RT (12 weeks, 3 times per week) contributed to an increase in MMP-2 activity in the biceps and gastrocnemius muscles of obese rats, which prevented the deleterious effects of a high-fat diet on skeletal muscle. Similarly, Prestes et al. (2009) observed an additional benefit of RT (12 weeks, 3 times per week) on MMP-2 activity in the soleus, tibialis anterior and extensor digitorium longus muscles in a model of hypoestrogenism. Thus, an increase in MMP −2 activity induced by RT has a positive biological effect.

In another study, Kim and Yi (2015) investigated the effects of single bout (30 min/day, 5 days/week for 6 weeks) and intermittent bouts (three times for 10 min/day, 5 days/week for 6 weeks) on MMP-2 expression in the extensor digitorum longus and soleus muscles of young and old rats. The exercise training was performed on a treadmill at a speed of 15 m/min (young) or 10 m/min (old) with a 5° incline. These authors demonstrated an increase in MMP-2 expression in all exercise groups when compared to control groups. However, Kim and Yi (2015) used different intensity, volume, type and duration of the training between young and old rats, which makes it difficult to compare their results to the results presented here. In addition, they assessed (Kim and Yi, 2015) MMP-2 using western blotting, while we analyzed activity MMP-2 using a zymography technique. This fact could also explain the discrepancy between the studies.

The sarcolemma in skeletal muscles has a basal lamina that provides structural support and maintains the physiological integrity of the myofibers and, moreover, has a major role in muscle fiber repair after RT (Carmeli et al., 2004). However, the collagen disorganization of the basal lamina in aging muscle promotes a reduction in the elasticity and endurance of myofibrils, which may compromise the power transmission from the tendons to the skeletal muscles (Kragstrup et al., 2011; Wood and Brooks, 2016; Zotz et al., 2016). In this respect, the increase in MMP-2 activity can indicate a positive modulation of physiological functions and tissue turnover in addition to contributing to favorable muscle fiber and connective tissue remodeling. Furthermore, MMP-2 is responsible for changes in the type IV collagen of the basal lamina, which is fundamental for joint movement, the passive elastic response of the muscle tissue in contractions (Pette, 2001) and the development of muscle strength after mechanical loading (Takala and Virtanen, 2000).

MMP-2 is also involved in the growth and development of myofibrils. Carmeli et al. (2004) reported that this enzyme is important for micro-trauma repair and releases local growth factors associated with the proteoglycan matrix (Tidball, 2005). In addition, MMP-2 can stimulate the proliferation, differentiation and migration of satellite cells to sites of injury, where they fuse to each other or to damaged fibrils, which allows for tissue regeneration (Yamada et al., 2008). However, the link between MMP-2 activity in muscle aging and state activation of satellite cells in response to RT remains to be determined.

On the other hand, it was observed that aging in sedentary animals also brought an increase in MMP-2 activity on the evaluated muscles. In aging, the inflammatory state influences the inhibition of cells responsible for hypertrophy, which contributes to sarcopenia. The age-related skeletal muscle loss results from multiple and complex proteolytic systems. It has been demonstrated that several inflammatory agents such as cytokines, advanced glycation end products and MMPs, together with storage inflammatory mediator cells (mast cells), are associated with inflammation (Payne, 2006). Thus, this mechanism may explain the high MMP-2 activity in the current study. Additionally, Barani et al. (2003) reported that protease expression maintenance during aging may ensure an adequate skeletal muscle response to harmful insults.

Another factor that should be considered is the point at which the old rats displayed higher body weight when compared to the young group. Previous data have already shown that the increased content of adipose tissue can also exert some negative influence on skeletal muscle remodeling (Souza et al., 2014). It has been demonstrated that the inflammatory profile associated with obesity contributes to pro-inflammatory cytokines that stimulate MMP-2 synthesis and activation, which compromise the lean mass and physiological function of obese rats (Souza et al., 2014). Most significantly, the current study suggests that RT can effectively prevent the deleterious effects of obesity associated with the aging process, since it induces improvements in ECM remodeling.

Interestingly, in our study there was no MMP-9 activity in the skeletal muscles. We only found it in the circulation of all the experimental groups and there was lower activity in the old animals. An important finding of the present study was that old rats demonstrated lower activity of MMP-2 and MMP-9 in blood circulation when compared to young rats. Gelatinase activity in circulation is important for aging, since it is associated with immunity, tumor progression and apoptosis (Nascimento et al., 2016). In human studies, higher levels of serum MMPs have been associated with obesity (Erman et al., 2016), aging (Nascimento et al., 2016), and pathological conditions such as type II diabetes with and without peripheral arterial disease (Signorelli et al., 2005), colorectal cancer (Tutton et al., 2003), and cardiovascular diseases (Posa et al., 2015). Previous investigations revealed that acute and chronic exercise modulates the MMP-2 and MMP-9 activity in blood circulation (Nascimento et al., 2015, 2016; Sousa Neto et al., 2017). In animal models, Posa et al. (2015) demonstrated that voluntary wheel-running exercise (6 weeks) induced decreased MMP-2 activity in blood circulation, improved the isolated heart perfusion and the ratio of infarct size, which can have beneficial effects for aging.

We might speculate that the aging process requires an accentuated modulation in skeletal muscle remodeling when compared with the young state due to possible weakness associated with the sarcopenia. Furthermore, RT can modulate the recruitment of MMPs from the circulation into skeletal muscles (Sousa Neto et al., 2017). These two explanations could justify the increased MMP-2 activity in muscles accompanied by a decrease in MMP-2 and MMP-9 activity in the blood plasma of old animals when compared to young rats. This possible explanation is consistent with Sousa Neto et al. (2017) who showed that high RT volume induced greater MMP recruitment to the gastrocnemius muscle when compared to low RT volume and a sedentary state. Furthermore, RT promotes a decrease in activity in circulation and adipose tissue due to increased blood flow to the gastrocnemius muscle, which required a greater demand for local remodeling. In addition, neutrophils and monocytes can migrate from the bloodstream into tissue that requires greater activity of MMPs, attributing to a decrease in MMP secretion by these cells in the blood (Newby, 2005).

Some limitations of the present study should be highlighted, such as the inability to analyze protein expression levels of MMP and inflammatory cytokines. In addition, the tissue inhibition of metalloproteinases (TIMPs) also modulates ECM remodeling, inhibiting the enzymatic functions of MMPs (Kim and Lee, 2016). The balance between MMPs and TIMPs must be well managed to optimize damage repair post exercise (Kim and Lee, 2016). There is little evidence to illuminate the relationship between MMPs and TIMPs in response to RT. Most of the existing studies have analyzed TIMPs in acute models and in blood circulation. Therefore, future studies should also investigate the ability of RT to modulate TIMPs in different skeletal muscles of old rats.

In conclusion, the present study proposes an additional mechanism to prevent the deleterious effects of aging. The results suggest that RT up-regulates MMP-2 activity in the gastrocnemius, flexor digitorum profundos, and soleus muscles accompanied by a down-regulation of MMP-2 and MMP-9 activity in the blood circulation of old rats.

## Author contributions

Conceived and designed the experiments: IdSN, JD, HdA, and RM. Performed the experiments: IdSN, JD, VG, and RM. Analyzed the data: IdSN, JD, VG, and RM. Contributed reagents, materials, analysis tools: IdSN, RT, JP, and HdA. Wrote the paper: IdSN, JD, RT, JP, and RM.

### Conflict of interest statement

The authors declare that the research was conducted in the absence of any commercial or financial relationships that could be construed as a potential conflict of interest.
